# Highlighting allelic variations at the interleukin-19 locus in term of preeclampsia predisposing factors and access to an accurate diagnostic/screening option

**DOI:** 10.1186/s12884-023-06143-x

**Published:** 2023-12-06

**Authors:** Sara Parhoudeh, Aida Saadaty, Khalil Khashei Varnamkhasti, Samire Khashei Varnamkhasti, Leila Naeimi, Sirous Naeimi

**Affiliations:** 1 Department of Genetics, College of Science, Kazerun Branch, Islamic Azad University, Kazerun, Iran; 2 Department of Medical Laboratory Sciences, Faculty of Medicine, Kazerun Branch, Islamic Azad University, Kazerun, Iran

**Keywords:** Preeclampsia, Genetic polymorphism, Interleukin-13, Interleukin-19, Pregnancy

## Abstract

**Background:**

Preeclampsia is the main cause of preterm parturition and maternal–fetal complications. T helper 1 and T helper 2 cytokines balance is a requirement in normal pregnancy and aberrant in this immunologic balance, play an important role in the pathology of preeclampsia. In previous studies single nucleotide polymorphisms have been associated with the alteration of serum cytokine levels.

**Objective:**

This study was aimed to discover association between interleukin-13 (rs20541, and rs56035208) and interleukin-19 (rs1028181 (T/C) and rs2243191(T/C)) polymorphisms with susceptibility to preeclampsia.

**Methods:**

In this case-control study 300 women with and without preeclampsia (*n* = 150/each) who referred to Zeynabieh Hospital- Shiraz, Iran, from February 2021 to April 2022 were enrolled. For genotyping the interleukin-13 and interleukin-19 polymorphisms, the Allele-specific polymerase chain reaction and direct sequencing method was carried out.

**Results:**

Our statistical results revealed no significant differences in allele and genotype frequencies for interleukin-13 polymorphisms compared to controls. We found that the interleukin‐13 polymorphisms are significantly associated with vulnerability to edema at rs20541 position and maternal drinking at rs56035208 position. But it was interesting to note that the differences of both the allele and genotype frequencies of interleukin-19 polymorphisms and their contribution to the risk of preeclampsia susceptibility were significant.

**Conclusions:**

No risk of preeclampsia was found in all comparisons for interleukin-13 polymorphisms. However, the interleukin-19 polymorphisms were found to confer the risk of preeclampsia in our population.

**Supplementary Information:**

The online version contains supplementary material available at 10.1186/s12884-023-06143-x.

## Introduction

 Preeclampsia is a common pregnancy-related disorder that affects 5–8% of all pregnancies [1]. The onset of this complication is after 20 weeks of gestation that originating at the maternal–fetal interface with obvious signs of multi-systemic involvement, from hypertensive disorders (systolic blood pressure (BP) ≥ 140 mm Hg or diastolic BP ≥ 90 mm Hg) and proteinuria (≥ 300 mg/24 h) till greater end-organ damage including hepatic alterations (hemolysis, elevated liver enzymes and low platelets (HELLP), placental structure and function impairment and subsequent increased oxidative stress and inflammation, pulmonary and neurological dysfunction including seizures [2–6]. Although understanding of mechanisms contributing to the pathophysiology of preeclampsia is an active area of international research, the etiology of preeclampsia remains unknown and except prophylactic use of low-dose aspirin (150 mg per day from 11 to 14 weeks of gestation until 36 weeks) in women who being identified at high risk for preterm preeclampsia, and preterm delivery that resolve signs and symptoms of preeclampsia no definitive treatment is available for preeclampsia [1, 7, 8]. Despite the exact causes of these alterations remain unclear, but it is well-established that unlike healthy pregnancy that is a controlled inflammatory process, preeclampsia is a proinflammatory state [8, 9]. Studies suggested a complex relationship between the pro-inflammatory condition of preeclampsia and immunologic aberrations. Evidence indicates that among different immune molecules, respective cytokines of T helper 1 (Th1) and T helper 2 (Th2) cells play an important role in controlling immune system function in various stages of pregnancy. In normal pregnancy, pro-inflammatory Th1 cytokines such as; interleukin 2, interferon gamma, transforming growth factor beta and angiotensin II type 1 receptor, which are involved in cellular immunity and mediate immune rejection of the fetus are decrease, by contrast, Th2 cytokines: interleukins 4, 5, 13 and 19 that involved in the maintenance of normal pregnancy occurrences, helps to neutralize pro-inflammatory cytokines, increased [10]. Imbalanced concentration of pro- and anti‐inflammatory cytokines likely contributes to the preeclampsia related complications [11]. Since the single nucleotide substitutions (SNPs) in the cytokine genes may affect cytokine transcription and influence its production [12], in our study, the association between preeclampsia and IL-13 gene rs20541 (G to A exchange) and rs56035208 (G to C exchange) polymorphisms and IL-19 rs1028181 (T to C exchange) and rs2243191(T to C exchange) have been assessed.

## Methods

### Study design

**Fig. 1:**
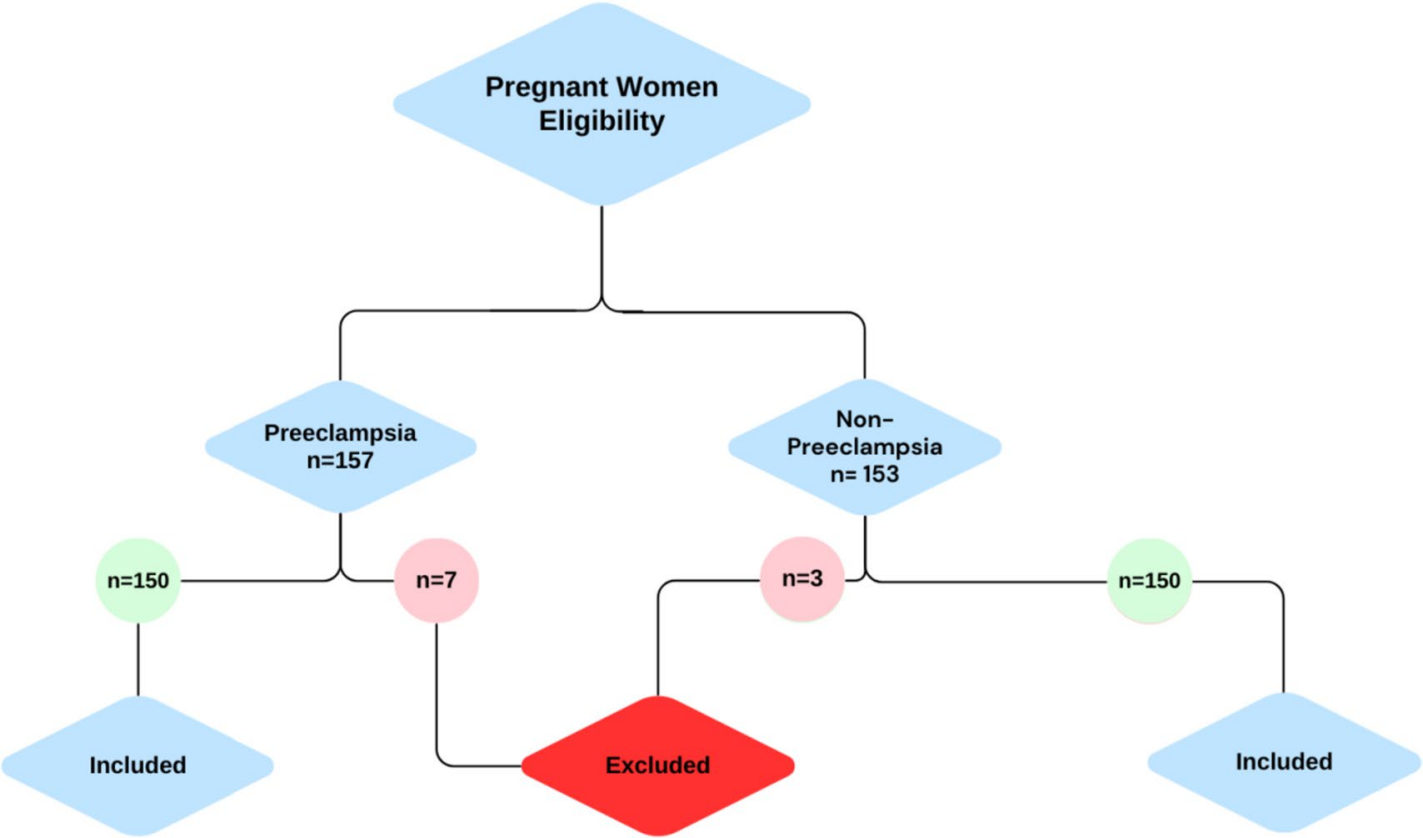
Flow chart of the selection procedure

In this case–control study, totally 300 participants, including 150 preeclamptic and 150 healthy pregnant women who were referred to Zeynabieh Hospital- Shiraz, Iran, during February 2021 to April 2022 enrolled. It is worth noting that this study conducted in a timeframe that a population under 1,000 of pregnant women had registered for medical care under the medical assistance insurance (Medicaid). Therefore, a minimum ratio of 30% (300 individuals) ensured representativeness of the sample. Demographic characteristics are shown in Table 1. The hallmark presentations of preeclampsia, blood pressure; systole > 140 mm Hg and diastole > 90 mm Hg on two occasions at least 4 h apart (the mild form of preeclampsia) or a shorter interval timing of systole > 160 mm Hg and diastole > 110 mm Hg (the severe form of preeclampsia) after 20 weeks’ gestation with accompanying proteinuria ≥ 300 mg upon a 24 h urine collection; or UPCR ≥ 0.3 mg/mg; or a urine protein dipstick reading ≥ 2+, were considered as definitive criteria for preeclamptic women. However, inclusion criteria were expanded by additional evidence of other significant findings that may be a part of the clinical presentation including end organ damage consisting of impaired liver function, severe persistent epigastric pain, new-onset headache, pulmonary edema, or renal insufficiency and abnormal lab values. Finally, women with high blood pressure accompanied with proteinuria, who have met the criteria of other organ involvement were included. The controls were collected from the healthy pregnant women, without hypertension, with the same gestational age (≥ 20 weeks), who had been referred to the midwifery clinic of the same hospital to receive prenatal care within the same timeframe. Pregnant women with a history of chronic hypertension and current antihypertensive treatment and any underlying disease such as renal, autoimmune, metabolic, liver, diabetic neuropathy or cardiovascular disease were excluded from our study (Fig. 1).

**Table 1:** Demographic characteristics of participants

Variables	Preeclampsia	Controls	*P*-value
	*N* = (150)	*N* = (150)	
Age (yr)	30.09 ± 6.87	27.23 ± 4.86	0.239
range	16–48	16–38	-
BMI (kg/m^2^)	26.19 ± 3.60	25.83 ± 3.47	0.214
Gestational age, median (range), wk (At the time of delivery)	34(32–37)	39(38–42)	-
Birth weight (Median)	2700	3000	0.309
Systolic blood pressure (mmHg)	151.84 ± 10.78	109.37 ± 11.07	< 0.001
Diastolic blood pressure (mmHg)	94.95 ± 6.88	64.85 ± 9.02	< 0.001

Significance level of values is 0.05. *P*-values were calculated with chi-square-test

### DNA extraction and genotype analysis

DNA was extracted from 5 ml peripheral blood, collected in EDTA tubes by Genomic DNA Isolation Kit (GeNet Bio, Daejeon, Korea), using salting out method. Allele-specific polymerase chain reaction (AS-PCR) (also known as amplification refractory mutation system (ARMS)) was designed for detection of rs20541 (G/A), rs56035208 (G/C), rs1028181 (T/C) and rs2243191(T/C) polymorphisms. For each variation, a pair of control primers specific for the normal DNA sequence (A pair of control primers which could not amplify mutant DNA at a given locus was used to confirm that the genomic DNA is, in principle, amplifiable) and allele-specific primers were designed using Oligo7 software (version 7.54, Molecular Biology Insights Inc., Cascade, CO, USA). (The designed primer sequences reported in Table 2).

**Table 2:** Designed primers for ARMS-PCR reactions and annealing temperature

SNP	Primer sequence	Annealing temperature (°C)
rs20541 (G/A)	F: CTTCCGTGAGGACTGAATGA RT: CTTTCGAAGTTTCAGTTGAACT RC: CTTTCGAAGTTTCAGTTGAACC F-PCR-Control: CCTCTGCACAGTTTGGAC R-PCR-Control: TCTGTCCAGCAATCCAGG	52 °C
rs56035208 (G/C)	F: TGTCAGGCGTCACCACTT RC: GACCAGCTCCTCAATGAGC RG: GACCAGCTCCTCAATGAGG F-PCR-Control: CCTCTGCACAGTTTGGAC R-PCR-Control: TCTGTCCAGCAATCCAGG	53 °C
rs1028181(T/C)	F: GCAAATGTGCTCAGTACTTG RT: CGT TTA ATC GCT CCT TAC AGT RC: CGT TTA ATC ATA GCT CCT TAC AGC F-PCR-Control: ATA TGG ATG CTT CAC ACA GAC C R-PCR-Control: TTC CCT GTA GTC AGG AAG	58.5 °C
rs2243191(T/C)	F: ACC TCA GGG AAG ATG T RT: CCT TGT CAT CAA GCT GAC A RC: CCT TGT CAT CAA GCT GAC G F-PCR-Control: CCT CTG CAC AGT TTG GAC R-PCR-Control: TCT CAG CAA TCC AGG	54 °C

**Fig. 2:**
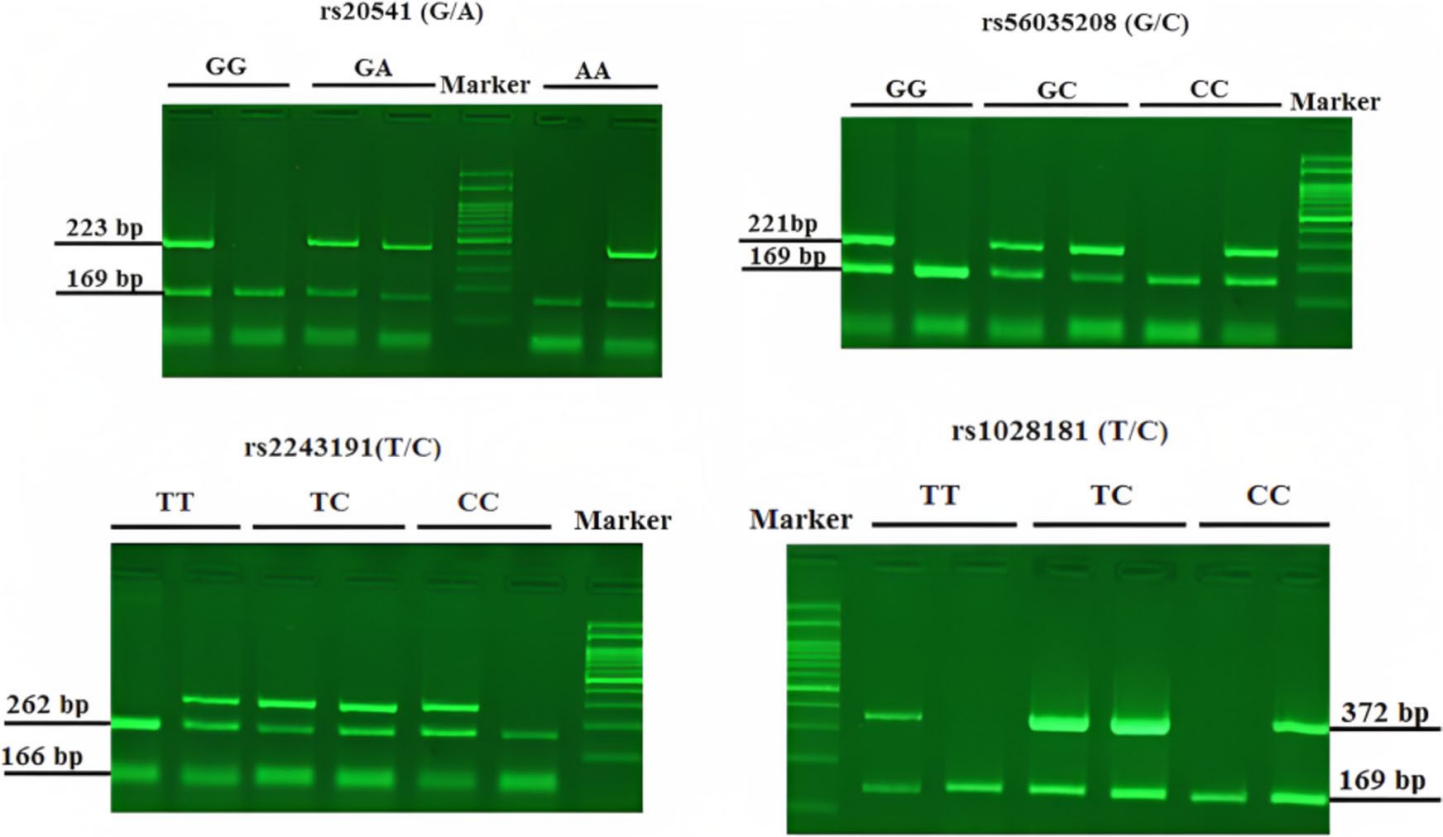
The pattern of bands observed after agarose-gel electrophoresis. IL-13; with control primers, a 169bp amplification product was obtained in two reactions. A 223bp (rs20541) and a 221bp (rs56035208) amplification products, are corresponding to the allele -specific primers used in AS-PCR. IL-19; with control primers, a 166 bp and a 169 bp amplification product were obtained in rs2243191 and rs1028181 reactions, respectively. A 262bp (rs2243191) and a 372bp (rs1028181) amplification products, are corresponding to the allele -specific primers used in AS-PCR. The gels displayed here not cropped, and are without high-contrast (overexposure)

PCR was performed in a total volume of 23.5 µl, including: 1 µl of template DNA, 12.5 µl of 2× Master Mix Red (Ampliqon), 1 µl (0/39 pmol) each primer, 5 µl H_2_O. The cycling conditions was as follows: first denaturation; 5 min at 94 °C, followed by 30 cycles at 94 °C for 40s (Denaturation), annealing temperature as in Table 2 for 40s and 1 min at 72 °C (Extension) and a final extension for 7 min at 72 °C. PCR products were run by standard electrophoresis on 2% agarose gel for 10 min and visualized on UV transilluminator. Bands length presented in Fig. 2.

Direct sequencing of PCR products recovered by the GEL/PCR Purification Kit (Favorgen Biotech Corp., Taiwan, China) was performed using Genetic Analyzer 3130x (Applied Biosystems, USA). Sequences were analyzed with the CodonCode Aligner V.5.1.5 software (CodonCode Corporation, Centerville, MA, USA).

### Statistical analysis

Data was analyzed by using, social sciences statistical software package for windows, version 19.0 (SPSS Inc, Chicago, Illinois, USA). The differences of the alleles and genotypes frequency were compared between preeclampsia and control groups by chi-square test. *P* value < 0.05 was considered statistically significant.

## Results

We first evaluated the Hardy–Weinberg equilibrium by computing expected genotype values versus observed genotype values for all polymorphic loci to check whether the population was in Hardy–Weinberg equilibrium. The results showed that the deviation from Hardy–Weinberg equilibrium in the all polymorphic loci were not significant, therefore equilibrium was maintained for in question population at polymorphic rs20541 (G/A), rs56035208 (G/C), rs1028181 (T/C) and rs2243191(T/C) sites.

**Fig. 3:**
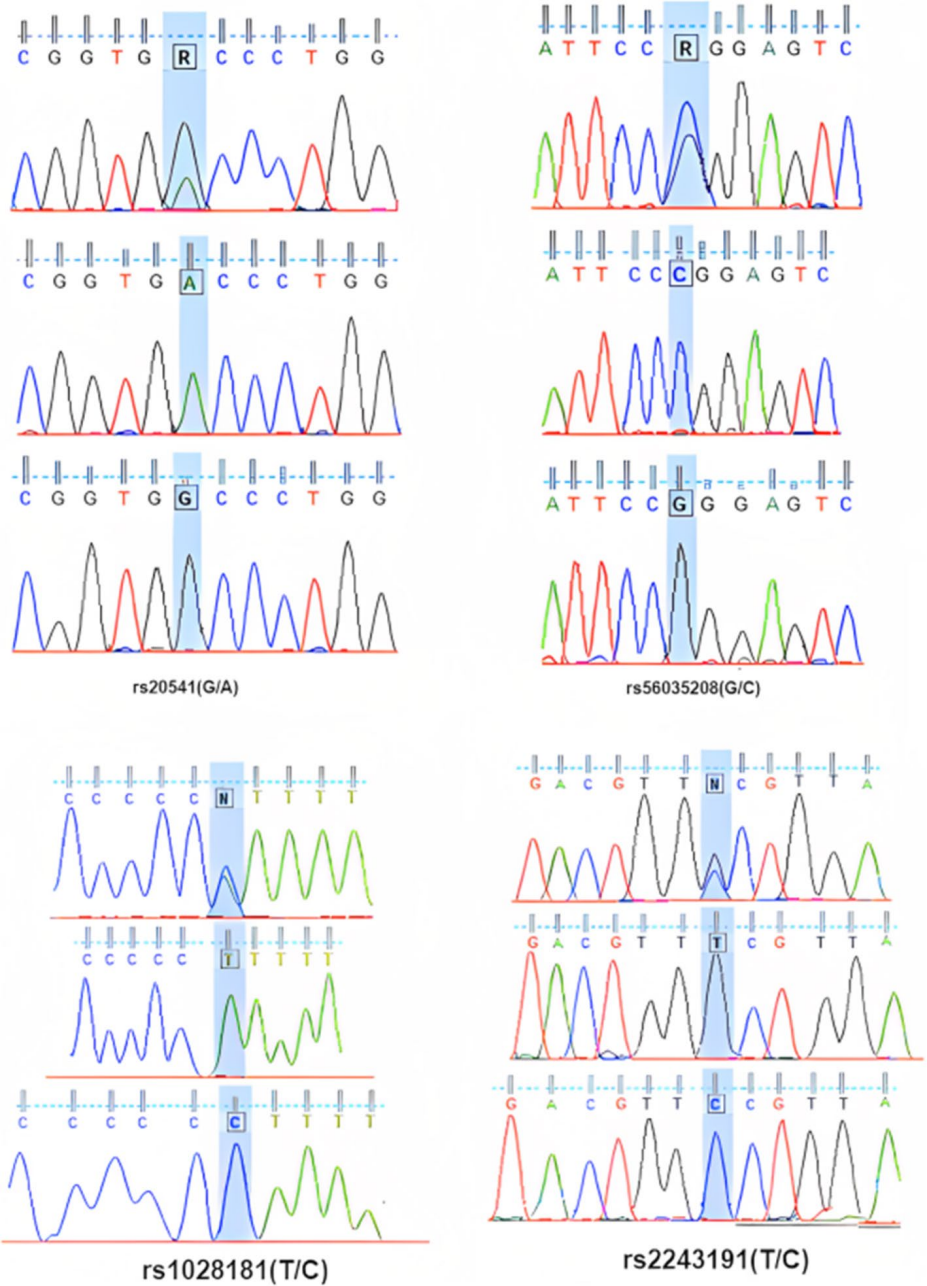
Result of DNA sequencing of PCR products of IL-13 and IL-19 polymorphisms

 The accuracy and specificity of our established AS-PCR was further validated by direct sequencing of PCR products. Figure 3 shows the sequencing result for samples.

As Table 3 displays the allele and genotype frequencies for IL-13 and IL-19 polymorphisms in the studied groups, no significant difference was observed in patients and controls groups allele (*P* = 0.8) and genotype (*P* = 0.4) frequencies for rs20541 (G/A) and allele (*P* = 0.2) and genotype (*P* = 0.6) frequencies of rs56035208 (G/C). The obtained results of the allele and genotype frequencies for the IL-19 gene SNPs showed statistically significant differences in comparisons of controls, with allele (*P* = 0.002), and genotype (*P* = 0.001) values for rs1028181, and allele (*P* = 0.042), and genotype (*P* = 0.032) values for rs2243191.

**Table 3:** Genotypes and alleles frequency of the IL-13 and IL-19 polymorphisms in preeclampsia and control groups

Genotypes and alleles	Control group	Patient group	*P*-value
	n (%)	n (%)	
rs20541 (G/A)			
GG	4(2.7)	1(0.7)	0.4
AA	3(2)	3(2)	
GA	143(95.3)	146(97.3)	
G	149(49.7)	152(50.7)	0.8
A	151(50.3)	148(49.3)	
rs56035208 (G/C)			
CC	146(97.3)	143(95.3)	0.6
GG	2(1.3)	4(2.7)	
CG	2(1.3)	3(2)	
C	6(2)	11(3.7)	0.2
G	294(98)	289(96.3)	
rs1028181 (T/C)			
CC	20(13.33)	23(15.33)	**0.001**
CT	31(20.66)	59(39.33)	
TT	99 (66)	68(45.33)	
C	71(23.66)	105(35)	**0.002**
T	229(76.33)	195(65)	
rs2243191(T/C)			
CC	94(62.67)	102(68)	**0.032**
CT	36(24)	41(27.33)	
TT	20(13.33)	7(4.67)	
C	224(74.67)	245(81.67)	**0.042**
T	76(25.33)	55(18.33)	

Data presented as n (%). Significance level of values is 0.05. *P*-values were calculated with chi-square-test.

To further identify a genetic risk for preeclampsia, we tested the association between preeclampsia risk markers such as seizure, proteinuria, edema, diabetes, multipara, hypertension, gravida, hyperthyroidism, HELLP syndrome, and history of abortion, history of preeclampsia, maternal drinking, maternal smoking, socioeconomic position, maternal BMI, birth weight and maternal age and rs20541 (G/A), rs56035208 (G/C), rs1028181 (T/C) and rs2243191(T/C) polymorphisms. In the case of IL-13 SNPs, except for edema at rs20541 position (*p* = 0.02) and maternal drinking at rs56035208 position (*p* = 0.002) no significant difference between the two groups for other risk markers observed (Table 4). Yet, the IL-19 SNPs were found to be associated with the more risk markers, such as; seizure (*p* = 0.005 _rs1028181_, 0.003 _rs2243191_), proteinuria (*p* = 0.023 _rs1028181_), edema (*p* = 0.017 _rs2243191_), diabetes (*p* = 0.023 _rs2243191_), hyperthyroidism (p = 0.015 _rs1028181_, 0.032 _rs2243191_), HELLP syndrome (*p* = 0.041 _rs2243191_), maternal smoking (*p* = 0.022 _rs1028181_) and maternal BMI (*p* = 0.045 _rs1028181_, 0.012 _rs2243191_). Significant risk markers are shown in Table 5.

**Table 4:** Association between the IL-13 (rs20541 (G/A) and rs56035208 (G/C)) polymorphisms and preeclampsia risk markers in preeclampsia and control groups

rs20541 (G/A)	Genotypes n (%)			*P*-value	rs56035208 (G/C)	Genotypes n (%)			*P*-value
	GG	AA	GA			CC	GG	CG	
Seizures Present	0 (0)	0 (0)	1 (0.7)	0.9	SeizuresPresent	1 (0.7)	0 (0)	0 (0)	0.97
Absent	1 (0.7)	3 (0.7)	145 (99.3)		Absent	142 (99.3)	4 (100)	3 (100)	
Absent	0 (0)	2 (67.7)	51 (34.9)	0.9	Proteinuria Absent	50 (0.35)	0 (0)	3 (100)	0.4
Trace	0 (0)	0 (0)	18 (12.3)		Trace	17 (11.9)	1 (25)	0 (0)	
**1**	1 (100)	1 (33.3)	44(30.1)		**1**	44 (30)	2 (50)	0 (0)	
**2**	0 (0)	0 (0)	15 (10.3)		**2**	15 (10)	0 (0)	0 (0)	
**3**	0 (0)	0 (0)	14 (9.6)		**3**	13 (9.1)	1 (25)	0 (0)	
**4**	0 (0)	0 (0)	4 (2.7)		**4**	4 (2/8)	0 (0)	0 (0)	
Edema Absent	0 (0)	1 (33.3)	22 (15.1)	**0.02**	Edema Absent	21 (14.7)	1 (0.25)	1 (33.3)	0.831
**1**	0 (0)	1 (33.3)	90 (61.6)		**1**	88 (61.5)	2 (50)	1 (33.3)	
**2**	0 (0)	1 (33.3)	24 16.4		**2**	23 (16.1)	1 (0.25)	1 (33.3)	
**3**	1 (100)	0 (100)	10 (6.8)		**3**	11 (7.7)	0 (0)	0 (0)	
Diabetes Present	0 (0)	0 (0)	9 (6.2)	0.8	Diabetes Present	9 (6.3)	0 (0)	0 (0)	0.9
Absent	1 (100)	3 (100)	137 (93.8)		Absent	134 (93.7)	4 (100)	3 (100)	
Multipara Primary	2 (40)	3 (50)	133 (46)	0.9	Multipara Primary	134 (46.4)	4 (66.7)	0 (0)	0.9
Multiple	3 (60)	3 (50)	156 (54)		Multiple	155 (53.6)	2 (33.3)	5 (100)	
Hypertension Mild	1 (100)	2 (66.7)	113 (77.4)	0.831	Hypertension Mild	111 (33.3)	2 (50)	3 (100)	0.831
Moderate	0 (0)	1 (33.3)	25 (17.1)		Moderate	25 (17.5)	1 (25)	0 (0)	
Severe	0 (0)	0 (0)	8 (5.5)		Severe	7 (4.9)	1 (25)	0 (0)	
Gravida Present	0 (0)	0 (0)	6 (4.1)	0.9	Gravida Present	6 (4.2)	0 (0)	0 (0)	0.9
Absent	1 (100)	3 (100)	140 (95.3)		Absent	137 (95.8)	4 (100)	3 (99.3)	
Hyperthyroidism Present	0 (0)	0 (0)	17 (11.6)	0.9	Hyperthyroidism Present	17 (11.9)	0 (0)	0 (0)	0.9
Absent	1 (100)	3 (100)	129 (88/4)		Absent	126 (88.1)	4 (100)	3 (100)	
HELLP syndrome Present	0 (0)	0 (0)	1 (7)	0.9	HELLP syndrome Present	1 (0.7)	0 (0)	0 (0)	0.9
Absent	1 (100)	3 (100)	145 (99.3)		Absent	142 (99.3)	4 (100)	3 (100)	
History of abortion Present	1 (100)	1 (33.3)	28 (19.2)	0.9	History of abortion Present	30 (21.7)	0 (0)	0 (0)	0.9
Absent	0 (0)	2 (66.7)	118 (80.8)		Absent	113 (79.3)	4 (100)	3 (100)	
History of preeclampsia Present	0 (0)	0 (0)	13 (8.9)	0.9	History of preeclampsia Present	12 (8.4)	0 (0)	1 (100)	0.9
Absent	1 (100)	3 (100)	133 (91.1)		Absent	113 (91.3)	4 (100)	2 (66)	
Maternal drinking Yes	1 (100)	0 (0)	0 (0)	0.062	Maternal drinking Yes	1 (100)	0 (0)	0 (0)	**0.002**
No	22 (14.8)	59 (39.6)	68 (45.6)		No	11 (7.4)	52 (34.9)	86 (57.7)	
Maternal smoking Yes	5 (38.46)	4 (30.76)	4 (30.76)	0.539	Maternal smoking Yes	10 (76.92)	3 (23.07)	0 (0)	0.63
No	64 (46.71)	47 (34.30)	26 (18.97)		No	92 (67.15)	38 (27.73)	7 (5.10)	
Socioeconomic position High	6 (75)	0 (0)	2 (25)	0.209	Socioeconomic position High	6 (75)	2 (25)	0 (0)	0.272
Middel	50 (43.10)	41 (35.34)	25 (21.55)		Middel	81 (69.8)	28 (24.1)	7 (6)	
Low	13 (50)	10 (38.46)	3 (53.11)		Low	15 (57.7)	11 (42.3)	0 (0)	
Maternal BMI <18.5	6 (54.54)	4 (36.36)	1 (9.09)	0.419	Maternal BMI <18.5	15 (60)	9 (36)	1 (4)	0.739
18.5–24.9	16 (64)	6 (24)	3 (12)		18.5–24.9	18 (78.26)	4 (17.39)	1 (4.35)	
25–29.9	39 (42.86)	31 (34.07)	21 (23.07)		25–29.9	60 (65.93)	26 (28.75)	5 (5.49)	
≥30	8 (34.78)	10 (43.48)	5 (21.74)		≥30	9 (81.82)	2 (18.18)	0 (0)	
Birth weight >2500 g	62 (43.97)	49 (34.75)	30 (21.27)	0.11	Birth weight >2500 g	96 (68.01)		7 (4.96)	0.75
							38 (26.95)		
<2500g	7 (77.77)	2 (22.22)	0 (0)		<2500g	6 (66.66)	3 (33.33)	0 (0)	
Maternal age <20	1 (12.5)	5 (62.5)	2 (25)	0.136	Maternal age <20	1 (12.5)	3 (37.5)	4 (50)	0.816
20–29	16 (13.8)	41 (35.3)	59 (50.9)		20–29	10 (8.6)	38 (32.8)	68 (58.6)	
≥30	6 (23.1)	13 (50)	7 (26.9)		≥30	1 (3.8)	11 (42.3)	14 (53.8)	

Data presented as n (%). Significance level of values is 0.05. *P*-values were calculated with chi-square-test.

**Table 5:** Association between the IL-19 (rs1028181 (T/C) and rs2243191 (T/C)) polymorphisms and preeclampsia risk markers in preeclampsia and control groups

rs1028181 (T/C)	Genotypes n (%)			*P*-value	rs2243191(T/C)	Genotypes n (%)			*P*-value
	CC	TT	CT			CC	TT	CT	
Seizures Present	1 (100)	0 (0)	0 (0)	**0.005**	Seizures Present	0 (0)	1 (100)	0 (0)	**0.003**
Absent	66 (43.7)	18 (10.3)	65 (46)		Absent	93 (60.8)	49 (33.8)	8 (5.4)	
Proteinuria Absent	9 (17)	20 (37.7)	24 (45.3)	**0.023**	Proteinuria Absent	4 (7.5)	18 (34)	31 (58.5)	0.056
Trace	2 (11.1)	7 (38.9)	9 (50)		Trace	1 (5.6)	6 (33.3)	11 (61.1)	
**1**	7 (15.2)	6 (40)	24 (52.2)		**1**	1 (2.2)	17 (37)	28 (60.9)	
**2**	1 (6.7)	10 (71.4)	8 (53.3)		**2**	3 (20)	4 (26.7)	8 (53.3)	
**3**	2 (14.3)	1 (25)	2 (14.3)		**3**	2 (14.3)	5 (35.7)	7 (50)	
**4**	2 (50)	15 (32.6)	1 (25)		**4**	1 (25)	2 (50)	1 (25)	
Edema Absent	3 (13)	11 (47.8)	9 (39.1)	0.093	Edema Absent	2 (8.7)	4 (17.4)	17 (73.9)	**0.017**
**1**	15 (16.5)	34 (37.4)	42 (46.2)		**1**	10 (11)	35 (38.5)	46 (50.5)	
**2**	3 (12)	11 (44)	11 (44)		**2**	0 (0)	10 (40)	15 (60)	
**3**	2 (18.2)	3 (27.3)	6 (54.5)		**3**	0 (0)	3 (27.3)	8 (72.7)	
Diabetes Present	2 (22.2)	2 (22.2)	5 (55.6)	0.054	Diabetes Present	0 (0)	5 (55.6)	4 (44.4)	**0.023**
Absent	31 (14.9)	57 (40.4)	63 (44.7)		Absent	12 (8.5)	47 (33.3)	82 (58.2)	
Hyperthyroidism Present	5 (29.4)	4 (23.5)	8 (47.1)	**0.015**	Hyperthyroidism Present	0 (0)	5 (29.4)	12 (70.6)	**0.032**
Absent	18 (13.5)	55 (41.4)	60 (45.1)		Absent	12 (9)	47 (35.3)	74 (55.6)	
HELLP syndrome Present	6 (20)	11 (36.7)	13 (43.3)	0.072	HELLP syndrome Present	13 (43.3)	14 (46.7)	9 (7.5)	**0.041**
Absent	17 (14.2)	48 (40)	55 (45.8)		Absent	9 (7.5)	39 (32.5)	72 (60)	
Maternal smoking Yes	3 (17.64)	11 (64.70)	3 (17.64)	**0.022**	Maternal smoking Yes	11 (64.70)	5 (29.41)	1 (5.88)	0.094
No	58 (43.60)	48 (36.09)	27 (20.31)		No	91 (68.42)	36 (27.06)	6 (4.51)	
Maternal BMI <18.5	13 (61)	3 (17)	5 (22)	**0.045**	Maternal BMI <18.5	0 (0)	19 (100)	0 (0)	**0.012**
18.5–24.9	68 (78)	9 (10)	14 (12)		18.5–24.9	11 (7.9)	70 (81)	13 (11.1)	
25–29.9	20 (72)	3 (24)	7 (4)		25–29.9	0 (0)	21 (93)	4 (7)	
≥30	4 (82)	2 (9)	2 (9)		≥30	3 (13.4)	7 (82.4)	2 (4.2)	
rs1028181 (T/C)	Genotypes n (%)			*P*-value	rs2243191(T/C)	Genotypes n (%)			*P*-value
	CC	TT	CT			CC	TT	CT	
Seizures Present					Seizures Present				**0.003**
	1 (100)	0 (0)	0 (0)	**0.005**		0 (0)	1 (100)	0 (0)	
Absent	66 (43.7)	18 (10.3)	65 (46)		Absent	93 (60.8)	49 (33.8)	8 (5.4)	
Proteinuria Absent	9 (17)	20 (37.7)	24 (45.3)	**0.023**	Proteinuria Absent	4 (7.5)	18 (34)	31 (58.5)	0.056
Trace	2 (11.1)	7 (38.9)	9 (50)		Trace	1 (5.6)	6 (33.3)	11 (61.1)	
**1**	7 (15.2)	6 (40)	24 (52.2)		**1**	1 (2.2)	17 (37)	28 (60.9)	
**2**	1 (6.7)	10 (71.4)	8 (53.3)		**2**	3 (20)	4 (26.7)	8 (53.3)	
**3**	2 (14.3)	1 (25)	2 (14.3)		**3**	2 (14.3)	5 (35.7)	7 (50)	
**4**	2 (50)	15 (32.6)	1 (25)		**4**	1 (25)	2 (50)	1 (25)	
Edema Absent	3 (13)	11 (47.8)	9 (39.1)	0.093	Edema Absent	2 (8.7)	4 (17.4)	17 (73.9)	**0.017**
**1**	15 (16.5)	34 (37.4)	42 (46.2)		**1**	10 (11)	35 (38.5)	46 (50.5)	
**2**	3 (12)	11 (44)	11 (44)		**2**	0 (0)	10 (40)	15 (60)	
**3**	2 (18.2)	3 (27.3)	6 (54.5)		**3**	0 (0)	3 (27.3)	8 (72.7)	
Diabetes Present	2 (22.2)	2 (22.2)	5 (55.6)	0.054	Diabetes Present	0 (0)	5 (55.6)	4 (44.4)	**0.023**
Absent	31 (14.9)	57 (40.4)	63 (44.7)		Absent	12 (8.5)	47 (33.3)	82 (58.2)	
Hyperthyroidism Present	5 (29.4)	4 (23.5)	8 (47.1)	**0.015**	Hyperthyroidism Present	0 (0)	5 (29.4)	12 (70.6)	**0.032**
Absent	18 (13.5)	55 (41.4)	60 (45.1)		Absent	12 (9)	47 (35.3)	74 (55.6)	
HELLP syndrome Present	6 (20)	11 (36.7)	13 (43.3)	0.072	HELLP syndrome Present	13 (43.3)	14 (46.7)	9 (7.5)	**0.041**
Absent	17 (14.2)	48 (40)	55 (45.8)		Absent	9 (7.5)	39 (32.5)	72 (60)	
Maternal smoking Yes	3 (17.64)	11 (64.70)	3 (17.64)	**0.022**	Maternal smoking Yes	11 (64.70)	5 (29.41)	1 (5.88)	0.094
No	58 (43.60)	48 (36.09)	27 (20.31)		No	91 (68.42)	36 (27.06)	6 (4.51)	
Maternal BMI <18.5	13 (61)	3 (17)	5 (22)	**0.045**	Maternal BMI <18.5	0 (0)	19 (100)	0 (0)	**0.012**
18.5–24.9	68 (78)	9 (10)	14 (12)		18.5–24.9	11 (7.9)	70 (81)	13 (11.1)	
25–29.9	20 (72)	3 (24)	7 (4)		25–29.9	0 (0)	21 (93)	4 (7)	
≥30	4 (82)	2 (9)	2 (9)		≥30	3 (13.4)	7 (82.4)	2 (4.2)	

Data presented as n (%). Significance level of values is 0.05. P-values were calculated with chi-square-test

## Discussion

Interleukin-13, that belongs to T helper 2 family and produced classically by T helper cells and also by eosinophils, basophils, mast cells, and NK cells, is a peptide cytokine coded by the gene *IL‐13* in the chromosomal location of 5q31.1, in a cluster close to *IL‐3*, *IL‐4*, *IL‐5*, and *CSF2* [13]. Since genetic factors are believed to be involved in the development of preeclampsia, and existing data confirm the association of *IL‐13* polymorphism with inflammatory states and disorders, e.g., association of SNP *IL‐13* + 1923 C/T with asthma development [14], or association of *IL13* rs20541 with significantly decreased susceptibility to renal cell carcinoma (a disease with immune predisposition) [15], our study aimed to analyze the possible impact of *IL-13* gene polymorphism with the susceptibility to preeclampsia. Two possible polymorphic sites of *IL-13* were identified, including rs20541 (G/A) and rs56035208 (G/C). The results revealed that none of the allele or genotype frequencies of *IL-13* rs20541 (G/A) and rs56035208 (G/C) polymorphisms had any significant association with preeclampsia susceptibility. Though very little is known about the *IL‐13* gene polymorphism on the development of preeclampsia, the impact of several other polymorphic sites have been reported. De Lima and colleagues did not find an association between polymorphisms in genes, *TNF-α* (− 308 G > A), *IL6* (− 174 G > C), *IFN-γ* (+ 874 A > T), *IL10* (− 1082 A > G), (− 819 C > T) and (− 592 C > A) and *TGF*- (+ 869 T > C) with preeclampsia [16]. In Pinheiro article also non-association between *TNF-α* (− 308 G → A), *IL-6* (− 174 G → C), or *IL-10* (− 1082 G → A) polymorphisms and preeclampsia was reported [9]. No significant difference in the distribution of genotypes and alleles of *IL-10* G-1082 A between the two groups was observed in the Sowmya study [17]. Tanaka and colleagues found no association between the rs4711998 A > G, rs8193036 C > T and rs2275913 A > G polymorphisms of the *IL17A* gene and the risk of developing preeclampsia [18]. In addition, findings of the current study, except edema in the position of rs20541 and maternal drinking at rs56035208 position, showed no significant association between other preeclampsia major risk factors include; seizure, proteinuria, diabetes, multipara, hypertension, gravida, hyperthyroidism, HELLP syndrome, history of abortion and history of preeclampsia, maternal drinking, maternal smoking, socioeconomic position, maternal BMI, birth weight and maternal age with rs20541 and rs56035208 polymorphisms. Similarly no significant relation in Lisi study, was found between preeclampsia risk factors, such as systolic and diastolic blood pressure, proteinuria and endothelin-1 type A receptor gene polymorphism (7231 G4A) [19]. Molvarec observed no significant relation between the TNF-alpha G-308 A polymorphism and HELLP syndrome [20]. PFAB and co-worker also reported that carrying C allele of the interleukin-6 polymorphisms (interleukin-6 S174G > C) significantly associated with new-onset edema in pregnant women [21].

On the contrary, the present study revealed association of *IL-19* gene polymorphisms in the preeclampsia group compared to the controls. Interleukin-19 is a member of the IL-10 family which is known as a pregnancy compatible cytokine [22]. The association of *IL-19* SNPs and preeclampsia has remained incompletely understood, but by increasing numbers of studies, strong evidence for the biological significance of *IL-19* thus far has been provided which indicates its significance in asthma [23–26], psoriasis [27], rheumatoid arthritis [28] and other immune diseases. Importantly, we have identified roles for *IL-19* SNPs as a genetic determiner that is associated with seizure, proteinuria, edema, diabetes, hyperthyroidism, HELLP syndrome, maternal smoking and maternal BMI during pregnancy.

Broadly speaking, new markers such as genetic characteristics, imaging, and biomarkers, are useful to improve risk prediction for patients and hold the promise of improving clinical prediction models. Based on the currently acknowledged risk factors, different professional organizations have thus far developed a number of prediction models for preeclampsia that in most models maternal serum PAPP-A and PlGF used as two biochemical predictors. Therefore, improving prediction models by maternal risk traits related markers (like maternal genetic variation considered in our study), with good discrimination and calibration, can show promising results in the early prediction of preeclampsia [29, 30]. Therefore, we conclude and recommend that IL-19 SNPs with high detection rates or low false-positive rates can be considered for improving prediction models at the time of model updating. As the available sample with the new marker in this study is small, our recommendation is using parsimonious methods for extending an existing prediction model from it that lead to the largest increase in discriminative ability of the prediction model [31].

Considering a larger sample size to evaluate association these in question polymorphisms with preeclampsia in future studies will resolve the present study limitation. Likewise, further studies on patients in a variety of ethnic populations are still required to increase our knowledge-base for these genes.

## Conclusion

Focused on polymorphisms and subtly investigating their potential role in preeclampsia is biologically or clinically important and might prove a reliable tool to identify and manage the women at risk.

### Supplementary Information

**Additional file 1.**

## Data Availability

The datasets generated and/or analysed during the current study are available in the [dbSNP] repository [http://www.ncbi.nlm.nih.gov/SNP]” and SNPs can be searched for using the dbSNP ID (rs20541, rs56035208, rs1028181and rs2243191).

## Abbreviations

IL-13: Interleukin‐13
IL-19: Interleukin‐19
Th1: T helper 1
Th2: T helper 2
BP: Blood pressure
SNP: Single nucleotide polymorphism
Wk: Week
Yr: Year
AS-PCR: Allele-specific polymerase chain reaction
HELLP: Hemolysis, Elevated Liver enzymes and Low Platelets
