# Supplementary figures and images for: Highlighting allelic variations at the interleukin-19 locus in term of preeclampsia predisposing factors and access to an accurate diagnostic/screening option
Source: BMC Pregnancy Childbirth. 2023 Dec 6;23:839. doi: 10.1186/s12884-023-06143-x (PMC10699059; doi:10.1186/s12884-023-06143-x)

**Original gels with visible membrane edges**


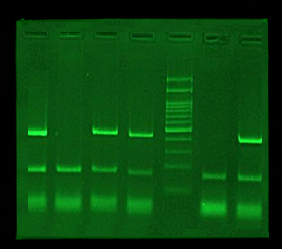


**rs20541**


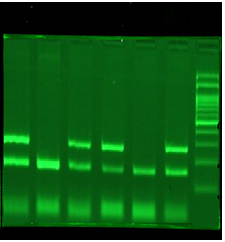


**rs56035208**


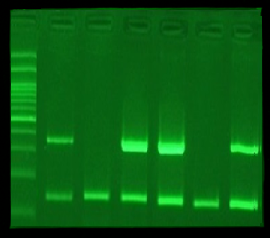


**rs1028181**


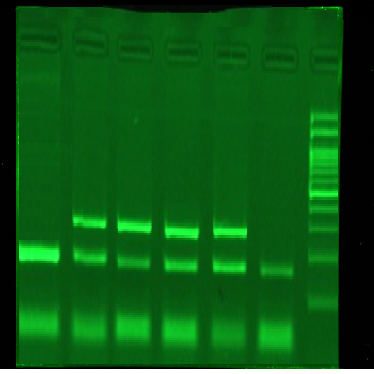


**rs2243191**

Supplement: Supplementary file 1 — Additional file 1. [file 12884_2023_6143_MOESM1_ESM.docx]
